# Craniofacial morphological variability in orthodontic patients with non-syndromic orofacial clefts: an approach using geometric morphometrics

**DOI:** 10.1007/s00784-024-05796-y

**Published:** 2024-07-02

**Authors:** Franca Schraad, Christian Schwahn, Karl-Friedrich Krey, Philine Henriette Doberschütz

**Affiliations:** 1 Department of Orthodontics, University Medicine Greifswald, Fleischmannstraße 42-44, 17475 Greifswald, Germany; 2 Department of Prosthetic Dentistry, Gerodontology and Biomaterials, University Medicine Greifswald, Fleischmannstraße 42-44, 17475 Greifswald, Germany

**Keywords:** Cephalometry, Cleft lip and palate, Cleft palate, Craniofacial morphology, Geometric morphometrics, Shape

## Abstract

**Objectives:**

Orofacial clefts are complex congenital anomalies that call for comprehensive treatment based on a thorough assessment of the anatomy. This study aims to examine the effect of cleft type on craniofacial morphology using geometric morphometrics.

**Materials and methods:**

We evaluated lateral cephalograms of 75 patients with bilateral cleft lip and palate, 63 patients with unilateral cleft lip and palate, and 76 patients with isolated cleft palate. Generalized Procrustes analysis was performed on 16 hard tissue landmark coordinates. Shape variability was studied with principal component analysis. In a risk model approach, the first nine principal components (PC) were used to examine the effect of cleft type.

**Results:**

We found statistically significant differences in the mean shape between cleft types. The difference is greatest between bilateral cleft lip and palate and isolated cleft palate (distance of means 0.026, *P* = 0.0011). Differences between cleft types are most pronounced for PC4 and PC5 (*P* = 0.0001), which together account for 10% of the total shape variation. PC4 and PC5 show shape differences in the ratio of the upper to the lower face, the posterior mandibular height, and the mandibular angle.

**Conclusions:**

Cleft type has a statistically significant but weak effect on craniofacial morphological variability in patients with non-syndromic orofacial clefts, mainly in the vertical dimension.

**Clinical relevance:**

Understanding the effects of clefts on craniofacial morphology is essential to providing patients with treatment tailored to their specific needs. This study contributes to the literature particularly due to our risk model approach in lieu of a prediction model.

## Introduction

Orofacial clefts are comparatively common congenital anomalies [1] with wide-ranging effects on craniofacial morphology, dentofacial relation and function [2–5]. This complexity and its substantial impact on both the childhood experience of the affected individual [6] and the quality of life for parents [7] call for comprehensive treatment plans based on a thorough assessment of the anatomical characteristics. Depending on the location, extent and laterality, orofacial clefts are classified into unilateral cleft lip, bilateral cleft lip, unilateral cleft lip and palate (UCLP), bilateral cleft lip and palate (BCLP), and isolated cleft palate (CP). The prevalence of these cleft types varies by sex [8, 9] and research indicates that specific cleft types are associated with characteristic craniofacial growth patterns and morphological changes [10–14].

Without surgical repair of the lip and palate, patients with BCLP often exhibit a prominent premaxilla and vertical growth pattern [15], while unoperated patients with UCLP may show a forward rotation of the premaxilla on the non-affected side, accompanied by a relatively retrusive maxilla and mandible [5], and unoperated patients with CP may show a retrusive maxilla and mandible relative to the cranial base [13]. Surgical repair of the lip and palate during childhood aims to influence craniofacial growth favorably, setting the stage for satisfactory aesthetic and functional outcomes in adulthood [16, 17]. But lip repair [18], an inadequate reconstruction of the nasolabial and perioral musculature [19], and the growth-restricting effects of scar tissue [20] can also impede craniofacial development. Recognizing this complex interplay, a comprehensive treatment approach must consider both internal and external factors that influence growth and function. By understanding how different cleft types react to surgical intervention, clinicians can refine existing treatment protocols to optimize patients’ outcomes. Prior research has primarily examined the craniofacial morphology of individuals with clefts in comparison with non-cleft individuals. To gain more insights into the craniofacial variations among different cleft types in adult patients, our study uses the methods of geometric morphometrics, a valuable tool to visualize this variability in an anatomical context [21, 22]. Unlike traditional morphometrics, which use the landmarks of cephalometric analyses to examine distances, angles, and ratios between them, geometric morphometrics focus on the overall shape formed by landmarks in relation to each other. The evaluation of lateral cephalograms of patients with cleft through geometric morphometrics therefore provides valuable insights into the complex craniofacial morphological changes caused by the condition [23].

Our aims were to use geometric morphometrics to examine: (1) the effect of cleft type on craniofacial shape (compared with sex); (2) whether variation in craniofacial shape increases with the extent of the cleft; and (3) assuming an effect of the cleft type on craniofacial shape, the specific anatomical regions where cleft types exhibit differences.

## Materials and methods

### Patients and data acquisition

We evaluated lateral cephalograms of 214 patients of Western European descent presenting with different types of non-syndromic, surgically repaired cleft formation. The clinical sample comprises all available and fully documented archived X-ray images used in our previous study [24], and corresponds to a catchment area of half of all individuals born with orofacial clefts in the former German Democratic Republic. All patients had been treated at the Wolfgang-Rosenthal Clinic Thallwitz (Germany), a specialized facility for the multidisciplinary treatment of patients with clefts. This ensured a large catchment area and high treatment standards. The patients had undergone late palate closure, meaning lip closure in the first six months of life and separate surgical repair of the palate in the fourth year of life. Patients had received orthodontic and orthopedic therapy; however, no orthognathic surgery had been conducted prior to the acquisition of the cephalometric X-ray images. All patients presented with cervical vertebral maturation stage of CS5 or CS6, indicating advanced stages of craniofacial growth [25]. Information on the chronological age of each patient was not available.

We digitized the X-ray films (4 m focus film distance, format 23.5 × 29.5 cm; digital format resolution: 300 dpi, gray shade: 16 bit, format: TIFF) using Intelli Scan 1600 (Quatographic Technology GmbH, Braunschweig, Germany). One investigator traced 16 hard tissue landmarks (Table 1) of the Bergen cephalometric analysis [26] on a high-resolution monitor (Barco Nio MDNC-2123, Barco, Kortrijk, Belgium) using Onyx Ceph dental imaging software (Image Instruments, Chemnitz, Germany). The reliability of landmark identification was assessed using repeated measurements taken eight weeks apart on 22 randomly selected patients (> 10% of total). All landmarks showed very good to excellent reproducibility [24]. Ethical approval for this retrospective evaluation of pseudonymized X-rays was obtained from the Scientific Ethical Committee of Greifswald University Medicine (Reg.-No. BB134/15).

**Table 1:** Description of landmarks. Landmarks are classified by their anatomical region to be used for shape visualization

Landmark symbol	Landmark	Description	Anatomical region for linked landmarks
N	nasion	most anterior point of nasofrontal suture	skull base
S	Sella	center of sella turcica	skull base
Cond	condylion	superior point of the condyle	mandible
Po	porion	most superior point of the external acoustic meatus	frankfort horizontal plane
Ba	basion	lowest and most posterior point of clivus	skull base
Ar	articulare	intersection of the lower edge of the skull base with dorsal contour of collum mandibulae	mandible
Me	menton	menton according to Hasund, lowest point on mandibular symphysis	mandible
Pg	pogonion	most anterior point of chin	mandible
Gn	gnathion	most anterior and inferior point of chin	mandible
B	B –point	most posterior point of anterior outline of mandibular alveolar ridge	mandible
ANS	anterior nasal spine	most anterior part of the nasal spine	maxilla
A	A-point	most posterior point of anterior outline of maxillary alveolar ridge	maxilla
Pm	pterygomaxillare	intersection of anterior border of ptergypalatine fossa with hard palate	maxilla
PNS	posterior nasal spine	most posterior part of the nasal spine	maxilla
Or	orbitale	most inferior point of the infraorbital ridge	frankfort horizontal plane
tGo	gonion tangent point	intersection between mandibular line and ramus line	mandible

### Cleft types

The patients were classified according to the LAHS nomenclature [27]. To compare the different cleft types and to account for the sample sizes of the resulting subgroups, we assigned the patients into three superordinate groups: BCLP, UCLP, and CP.

### Geometric morphometrics: shape and size

**Fig. 1:**
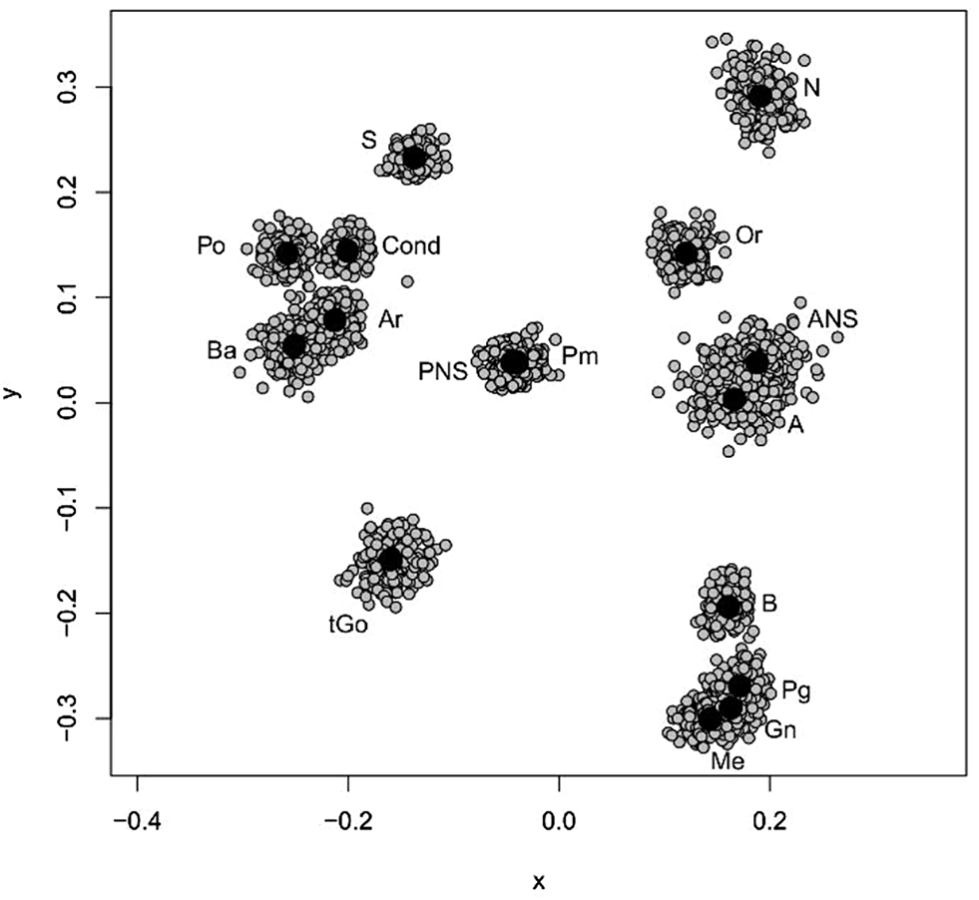
Procrustes shape coordinates of all individuals (gray) and of the mean shape (black)

We performed a Generalized Procrustes analysis by centering the landmark coordinates to the origin, and translating, rotating, and scaling them to centroid size until the sum of squared distances among homologous landmarks was minimized [28]. The centroid size is a unit measure of size and is computed as the square root of the sum of squared distances of all landmarks from the centroid [29]. This adjustment allows the resulting Procrustes shape coordinates (Fig. 1) to be used for further shape analysis.

### Design options used to prevent confounding

To quantify the total effect [30, 31] of cleft types on morphological shape and following the theory of causal inference [30–32], we did not include pure predictors in the risk model but only those confounders that constitute an adequate confounding set [32]. For that, we modelled directed acyclic graphs (DAGs) [31] for the exposure cleft type, the outcome shape, the confounder sex, the variable size, and an unknown variable affecting cleft type and size. The confounder sex meets the three criteria for a single confounder [31, 33], whereas the variable size does not because size does not temporally precede the manifestation of an orofacial cleft.

We first examined the simple DAG model including sex and size as potential variables of a confounding set, with arrows from sex going to cleft type, size, and shape and an arrow from size only going to shape, as explained above, by using DAGitty [34, 35]. This DAG model has only a single minimal sufficient adjustment set (confounding set) consisting solely of sex. The simple DAG model, however, has a testable implication, namely that cleft type is independent of size when conditioning on sex [34]. Using Stata software (Stata Corporation, College Station, TX, USA; release 17.0), this implication was tested in a multinomial logistic regression with the categorical variable cleft type as the dependent variable, and sex and size as independent variables. The simple DAG model was accepted (*P* > 0.7926).

We additionally assumed an unknown variable (which might be a genetic factor) from which arrows point to cleft type and size (but not to shape) [32, 34]. For this expanded DAG model, two minimal sufficient adjustment sets, including sex and either size or the unknown variable, are available. Declaring size instead of cleft type as exposure in this expanded DAG model, the resulting adjustment set of sex and cleft type for the relation between size and shape leads to the same variables in the final model: sex, size, and cleft type. Since the expanded DAG model has no testable implications without the unknown variable, we considered sex and size as the adjustment set in the main analysis.

For the relation between sex and shape with sex as the exposure of interest, we did not adjust for size, cleft type, or both. Hence, the total effects of sex are unadjusted unless stated otherwise.

Our variable selection procedures accounts for the often overlooked difference between predictive models and causal inference models [30]. Besides variable selection procedures to differentiate the risk model from predictive models, the outcome shape (defined by the selected landmarks) can differ in risk and prediction models of shape, which is pronounced in selecting landmarks in the affected cleft area.

### Statistical analysis

The centroid size was graphed using a box plot stratified by sex and, in a linear regression framework for morphometric analysis, modelled by sex and cleft type [36]. We performed a multivariate regression to assess the possible effects of sex, size, and cleft type on shape. Shape differences between sexes and cleft types were analyzed using pairwise comparisons. Thereby, pairwise distances between mean landmark configurations (calculated as least-square means) and between variances (as the mean dispersion of values) were computed, estimated, and tested. Shape differences were visualized by displaying the mean shapes with landmark links.

We used a principal component analysis (PCA) of the Procrustes shape coordinates to depict the most important aspects of the multivariate data set in two dimensions: the original variables were recombined into linear variables (principal components, PC) to explain as much variance in the multivariate data set as possible [37, 38]. In prediction models, it is well justified to focus on the first two PCs, which cover the two largest variances of shape. As this is a common procedure in similar studies, we briefly examined PC1 and PC2 for comparative purposes. In risk models, however, it is not reasonable to expect that the exposure has a relevant effect on the first two PCs. Therefore, we used the following approach suitable for risk modelling of the outcome shape: provided that the cleft type affects shape in a multivariate shape analysis, we used the first principal components (which together explain at least 80% of the variance) and examined the exposure effect on single PCs in separate regression analyses. We illustrated the PCs on which the exposure had the greatest influence. For better visualization, shape changes along the axes of the different principal components were illustrated using thin-plate spline to create transformation grids with additional landmark links [22].

For shape analyses we used the R package “geomorph”, version 4.0.4 [36, 39, 40]. The statistical significance of the variance analyses was tested with permutation methods using 10,000 iterations, with a level of 95% for upper confidence limits (UCL) [41]. We used the “rms” package for the analysis of shape represented by a PC as the dependent variable in the univariate regression analysis [42]. To address the most important mathematical assumption of the linear regression model (ordinary least squares regression), we modelled departures from linearity for size by modelling restricted cubic splines with 3 knots requiring 2 degrees of freedom [42, 43]. Natural heterogeneity of residuals in ordinary least squares regression was adjusted for by applying Efron’s robust variance estimator [42]. All artwork was created with R Studio. [40]

## Results

### Relations among cleft type, sex, and size

**Fig. 2:**
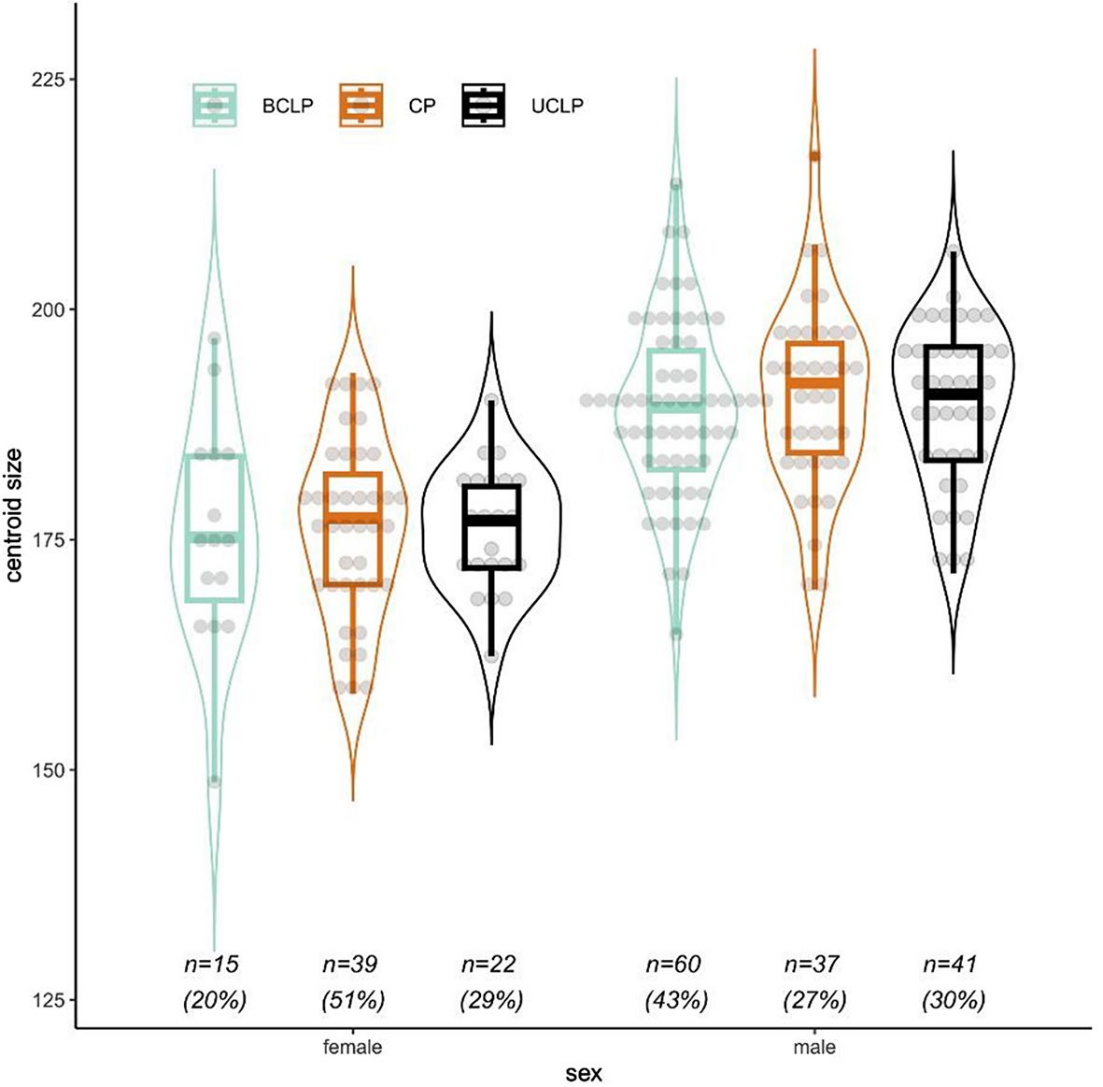
Box and violin plot of centroid size. The plot shows the centroid size distribution for BCLP (green), CP (orange) and UCLP (black) stratified by sex

Lateral cephalograms of 76 female patients and 138 male patients were available for analysis: 75 patients presented with BCLP, 63 patients with UCLP, and 76 patients with CP. The proportion of BCLP patients is higher among males than among females, and the opposite is true for CP (Fig. 2) (*P* = 0.0003 for *Χ*² test with 2 degrees of freedom). Size substantially depends on sex but not on cleft type (Fig. 2) (*P* = 0.0001 and *P* = 0.7923, R² = 0.316 and R² = 0.001, respectively, in Procrustes regression).

### Effect of cleft type on shape (compared with sex)

Cleft type has a larger effect on shape than sex has, but a smaller effect compared with size (partial R² = 0.012, partial R² = 0.026, and partial R² = 0.039; *P* = 0.0189, *P* = 0.0012, and *P* = 0.0001 for sex, cleft type, and size, respectively, Procrustes regression).

To assess the total effect of cleft type, we compared the pairwise distances of variances and means for cleft types and sex. The distance between variances of females and males is 0.0009 (females 0.0057, males 0.0066, 95% UCL = 0.0010; *P* = 0.0691, Procrustes regression). While the variances in CP and UCLP are similar to that in females, the variance in BCLP is similar to that in males (Table 2). Correspondingly, BCLP differs from CP and UCLP. The distance between means of females and males is 0.0180 (95% UCL = 0.0161; *P* = 0.0189), whereas the point estimate for distances between cleft types is greater than 0.0210 (Table 2).

**Fig. 3:**
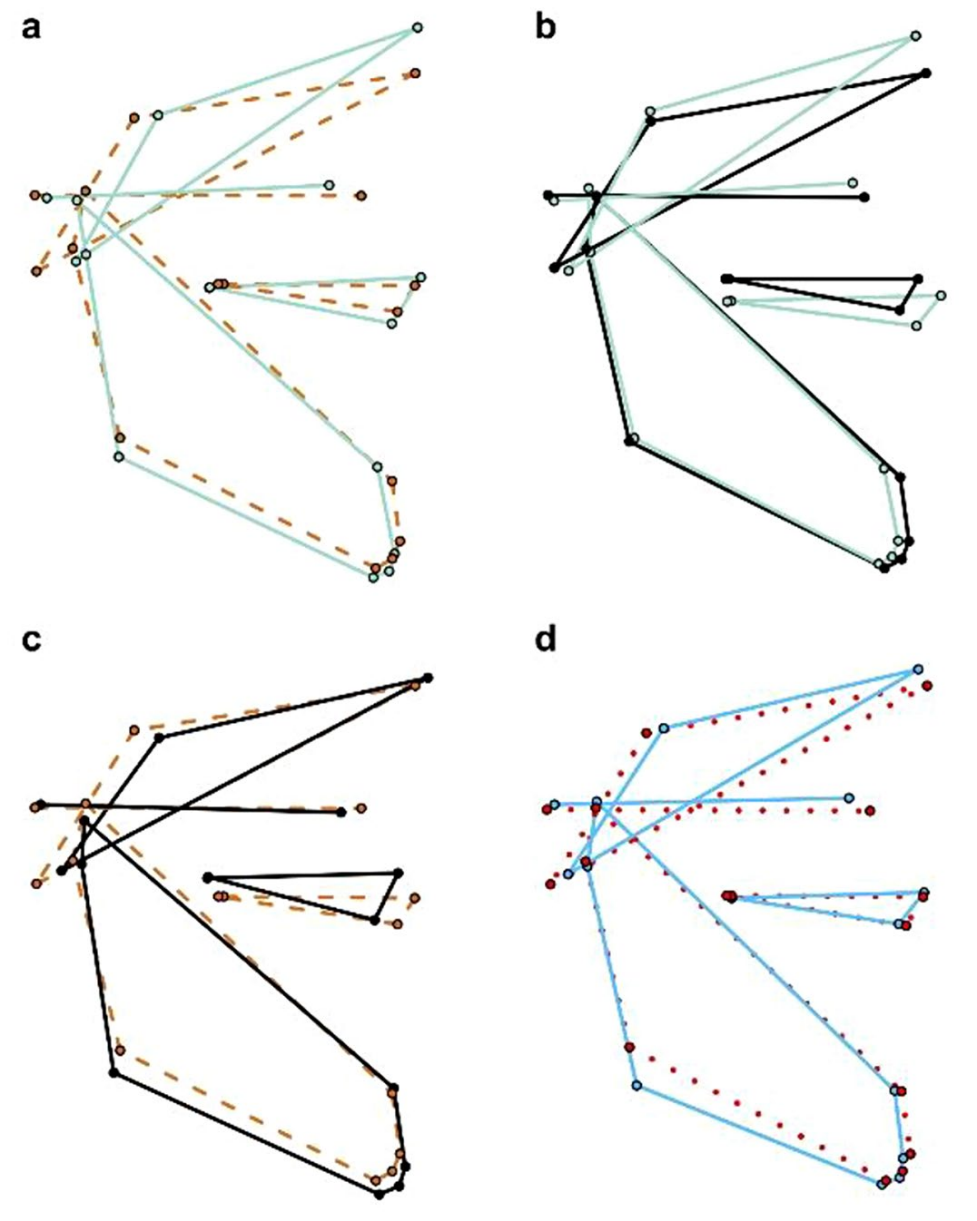
Wireframes showing the differences between the mean shapes of cleft types and sex. (**a-c)** show the difference between the mean shapes of BCLP (solid green line), CP (dashed orange line) and UCLP (solid black line). (**d)** shows the difference between the mean shapes of females (dotted red line) and males (solid blue line)

The change in the coefficients of interest (distances of variances and means for comparison between cleft types) is less than 10% by including the potential confounders sex and size (Table 2), meaning that plots of the observed mean shapes of cleft types can be interpreted directly (Fig. 3). To better visualize shape differences, we magnified the mean shapes four times and linked landmarks (Table 1).

**Table 2:** Variances, distances of variances, and distances of means for cleft types

Cleft type or comparison	Not adjusted	Adjusted for sex	Adjusted for sex and linear size	Adjusted for sex and nonlinear size	
		**Variances**			
BCLP	0.00719	0.00712	0.00689	0.00682	
CP	0.00575	0.00570	0.00549	0.00544	
UCLP	0.00548	0.00546	0.00527	0.00527	
		**Distances of variances** (95% UCL)			*P value*
BCLP - CP	0.00145 (0.00111)	0.00142 (0.00109)	0.00140 (0.00101)	0.00138 (0.00100)	*0.0060*
BCLP - UCLP	0.00172 (0.00115)	0.00166 (0.00114)	0.00162 (0.00107)	0.00155 (0.00106)	*0.0032*
CP - UCLP	0.00027 (0.00112)	0.00024 (0.00112)	0.00022 (0.00104)	0.00017 (0.00105)	*0.7494*
		**Distances of means** (95% UCL)			*P value*
BCLP - CP	0.02609 (0.01841)	0.02609 (0.01950)	0.02624 (0.02121)	0.02609 (0.01934)	*0.0011*
BCLP - UCLP	0.02183 (0.01933)	0.02183 (0.01952)	0.02255 (0.01961)	0.02183 (0.01925)	*0.0155*
CP - UCLP	0.02344 (0.01941)	0.02344 (0.01960)	0.022612 (0.01989)	0.02344 (0.01925)	*0.0067*

#### BCLP compared with CP

Patients with BCLP show a longer ascending part of the mandible and a relatively longer upper face (N-ANS) than those with CP. In patients with BCLP, the mandible and the maxilla are positioned more posterior relative to the skull base than in patients with CP (Fig. 3a).

#### BCLP compared with UCLP

Patients with BCLP show a longer upper face (N-ANS) and a shorter lower face (ANS-Me) than those with UCLP. In patients with BCLP, the maxilla is positioned more anterior relative to the mandible than in patients with UCLP (Fig. 3b).

#### UCLP compared with CP

Patients with UCLP show a longer ascending part of the mandible than those with CP. In patients with UCLP, the mandible is positioned more posterior relative to the skull base and more anterior relative to the maxilla than in patients with CP (Fig. 3c).

#### Females compared with males

Sexual dimorphism manifests itself only in slight differences: male patients show a longer ascending part of the mandible and a smaller angle of the mandible (Fig. 3d).

### Results of the principal component analysis

**Fig. 4:**
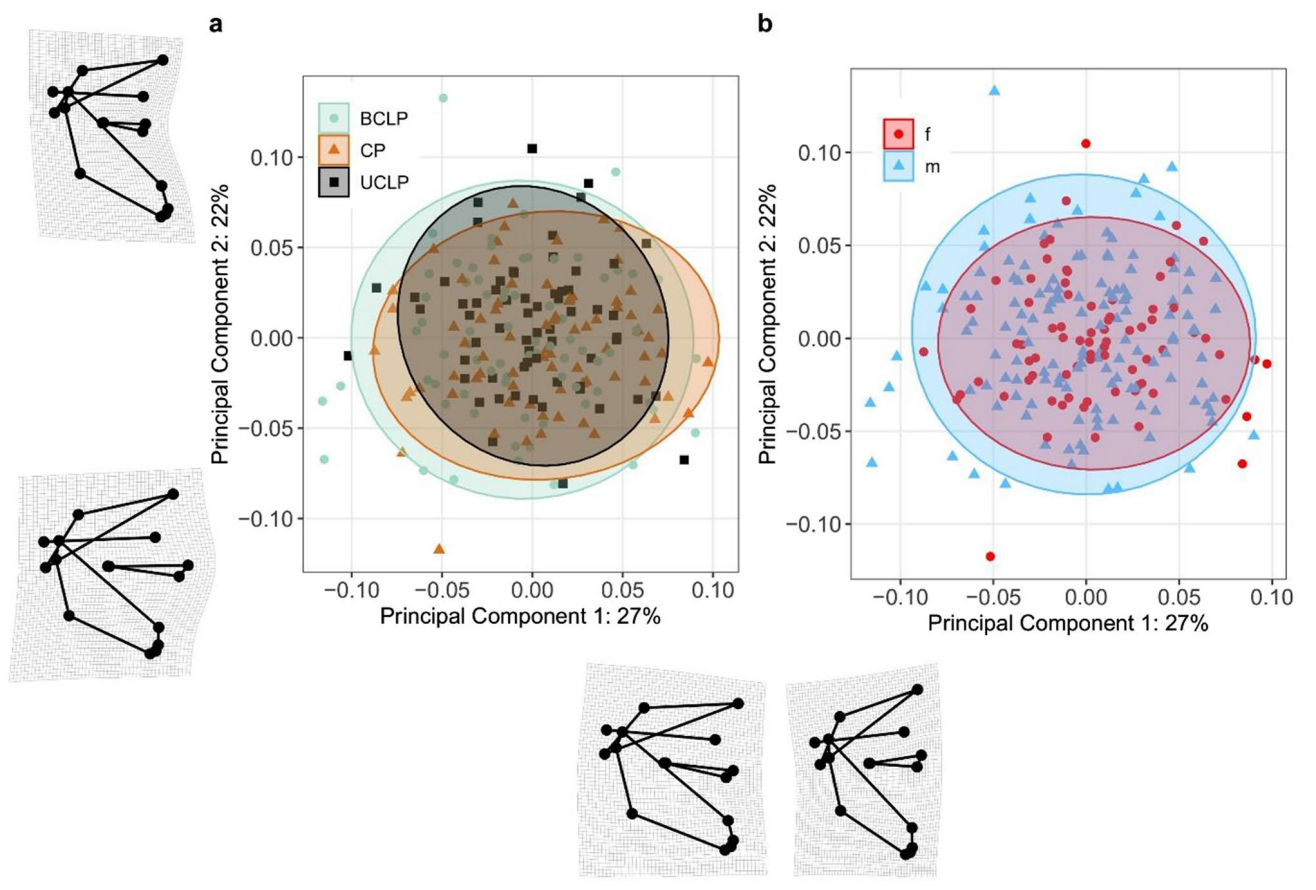
Scatterplot of the first two principal components. (**a)** scatterplot with individuals marked by cleft type. (**b)** scatterplot with individuals marked by sex. Extreme shapes of positive and negative values of PC1 and PC2 are represented by transformation grids. The corresponding coordinates are: PC1 min (-0.116, -0.035), PC1 max (0.097, -0.014), PC2 min (-0.052, -0.012) and PC2 max (-0.049, 0.133)

The plot of PC2 against PC1 shows overlapping ellipses (including 95% of the points), indicating limited overall variation in individual shapes (Fig. 4). PC1 and PC2 jointly account for 49% of the total shape variation, with PC1 accounting for 27% and PC2 accounting for 22%. Shape alters along the PC1 and PC2 axes: PC1 mainly shows shape differences in the vertical dimension of the anterior facial height compared with the almost unchanged posterior facial height. PC2 mainly shows shape differences in the sagittal plane, especially regarding the anteroposterior relation of the maxilla to the mandible. Neither PC1 nor PC2 can depict the effect of cleft types on shape as found in the multivariate analysis, thus warranting separate univariate regression analyses.

**Fig. 5:**
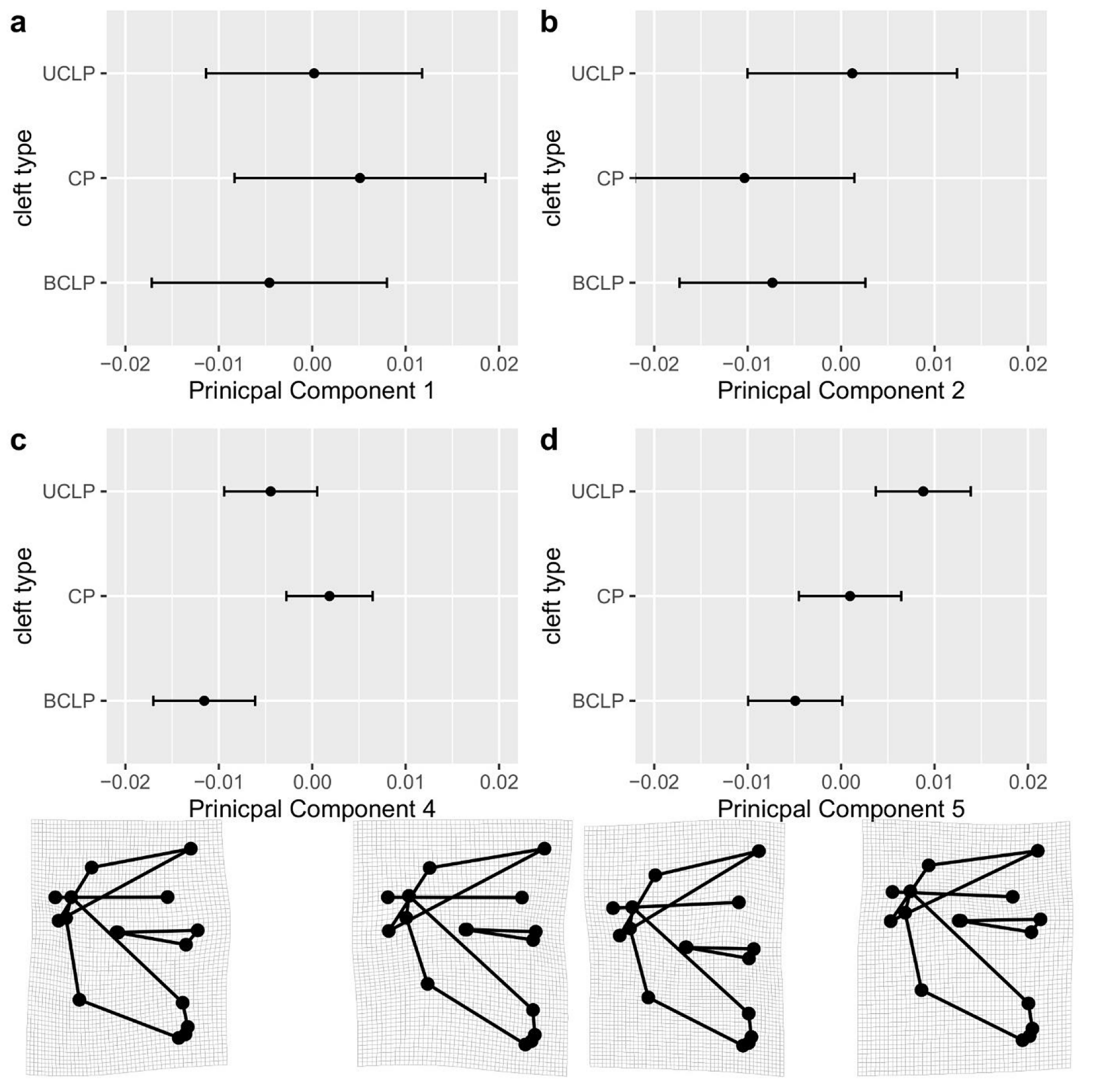
Effect of cleft type on PC1, PC2, PC4 and PC5 (**a-d**) with transformation grids for PC4 min (-0.052, -0.003), PC4 max (0.048, 0.01), PC5 min (0.017, -0.063) and PC5 max (-0.001, 0.051)

The first nine PCs together explain 83% of the variance in Procrustes PCA of shape data (Table 3). Differences among cleft types are most pronounced for PC4 and PC5 (Table 3). Although effects on PC9, PC8, and PC7 (but not PC3) are statistically significant on an alpha level of 5% after correction for multiple testing by using Holm’s procedure, we primarily interpret effect sizes as recommended by Wasserstein and Lazar [41] and present only the plots for PC4 and PC5 with transformation grids (Fig. 5). The effect of cleft type was adjusted for sex and size, whereas conventional plots for PC1 and PC2 on x and y axis, respectively, are based on observed data.

PC4 accounts for shape variation in the mandible and maxilla: PC4min, where the UCLP and especially the BCLP group tend to be located, shows a greater posterior height of the mandible, a smaller angle of the mandible and a more pronounced maxilla, compared with PC4max, where the CP group tends to be located (Fig. 5c). BCLP, CP and UCLP (in this order) spread along the axis of PC5 from negative to positive values. Shape changes show a longer upper anterior facial height in relation to a shorter lower anterior facial height for PC5min, and a reversed relationship for PC5max (Fig. 5d).

Shape changes illustrated along the axes of PC4 and PC5 match those seen between the mean landmark configurations.

**Table 3:** Univariate regression analysis of PC1-PC9 on sex, size, and cleft type

Outcome used in univariate regression: PC of PCA for multivariate shape				Total effect of sex		Independent variables of the final model; acc. to DAGs, total effects are those for cleft type and size, but not for sex								
PC	Proportion of variance in PCA	Cumulative proportion in PCA		Sex		Sex		Size		Cleft type		Regression		
	%	%	SD	R²	*P*	partial R²	*P*	partial R²	*P*	partial R²	*P*	R²	*P* for joint test	
PC 1	27.0	27.0	0.0416	0.008	0.1938	0.011	0.1294	0.014	0.2286	0.008	0.4288	0.032	0.1826	
PC 2	22.2	49.2	0.0377	0.002	0.4546	0.027	0.0125	0.085	0.0001	0.017	0.1420	0.111	0.0002	
PC 3	8.2	57.5	0.0229	0.005	0.3211	0.029	0.0110	0.056	0.0021	0.029	0.0373	0.076	0.0012	
PC 4	5.7	63.2	0.0192	0.106	< 0.0001	0.009	0.1081	0.033	0.0110	0.073	0.0001	0.220	< 0.0001	
PC 5	5.1	68.3	0.0180	0.002	0.5490	0.001	0.6289	0.005	0.5836	0.091	< 0.0001	0.108	0.0006	
PC 6	4.1	72.4	0.0162	0.010	0.1622	0.004	0.3525	0.000	0.9942	0.004	0.6726	0.013	0.7143	
PC 7	3.8	76.2	0.0155	0.003	0.3960	0.001	0.6941	0.002	0.7790	0.051	0.0040	0.054	0.0332	
PC 8	3.4	79.6	0.0148	0.006	0.2382	0.013	0.0904	0.000	0.9633	0.052	0.0038	0.062	0.0346	
PC 9	3.1	82.7	0.0140	0.016	0.0716	0.003	0.4338	0.024	0.0638	0.063	0.0010	0.112	0.0012	

Abbreviations DAG directed acyclic graph, PC Principal Component, PCA Principal Component Analysis, SD standard deviation

## Discussion

We found statistically significant differences in the mean shape between all three cleft groups, suggesting that the cleft type influences the craniofacial shape. The difference is greatest between BCLP and CP – the most and least extensive cleft defects in our sample, respectively. Patients with BCLP and UCLP show some similarity in the mean shape. Other studies using geometric morphometrics have compared patients with cleft with non-cleft control groups, but confirm the general trend: Bugaighis et al. have found that the BCLP and UCLP groups differ the most from a control group of non-cleft children, whereas the CP group differs the least from the non-cleft control group [23]. Toro-Ibacache et al. have found statistically significant differences between the mean shape of adults with UCLP and a non-cleft control group [44]. Latif et al. have confirmed differences between the mean shapes of unrepaired BCLP and a non-cleft control group [45]. The effect of cleft type is greater than the effect of sex on shape. Latif et al. have found no sexual dimorphism in subjects with unrepaired BCLP [45].

The variation in shape increases with the extent of the cleft: there is more variation in the BCLP group, and less in the UCLP group and CP group. Our results show clearly overlapping scatterplots for PC2 plotted against PC1, indicating limited overall variation in individual shapes. Latif et al. have shown overlapping scatterplots for unrepaired BCLP and a non-cleft control group [45]. Bugaighis et al. and Toro-Ibacache et al. have reported some overlap in the scatterplot of PC2 against PC1 for CP compared with a non-cleft control group, and UCLP compared with a non-cleft control group, respectively [23, 44]. Comparability with these studies is limited because we compare the cleft types with each other and not with a control group of unaffected individuals.

The anatomical regions in which the cleft types differ are mainly in the vertical dimension: the wireframes of the mean shapes show shape differences in the ratio of the upper face to the lower face, the posterior height of the mandible, and the mandibular angle. The extremes of PC4 and PC5 underline these differences. We found a longer upper face in patients with BCLP compared with the UCLP and CP group. Considering that BCLP is a more extensive cleft defect than UCLP and CP, our findings appear to be a continuation of the results of Toro-Ibacache et al. and van den Dungen et al., who have described a vertically elongated face in patients with UCLP and BCLP, respectively, compared with non-cleft control groups [4, 44]. The underlying clockwise rotation of the mandible has been attributed to a narrow nasal cavity after palatal surgery leading to increased mouth breathing [46] and to a low tongue position in patients with clefts [47]. The longer upper face in patients with BCLP may result from an additional clockwise rotation of the maxilla due to scarred tissue in the palatal region as well as surgical lip repair [48].

Our results show small changes in the anterior-posterior direction. Based on our findings, it can be hypothesized that the initial growth dynamics of the premaxilla before lip surgery play a pivotal role in subsequent craniofacial development. In patients with BCLP, the premaxilla experiences unrestrained forward growth before or without surgical intervention [15, 45, 49, 50]. After repair, however, excessive lip pressure can restrain maxillary forward growth [18, 51, 52]. A prior anterior positioning of the maxilla may provide an advantage against the growth-restraining effects of scar tissue following lip repair surgery. This could explain our finding that compared with patients with UCLP, patients with BCLP show a less posterior maxilla relative to the mandible and skull base. Compared with patients with CP, who are unaffected in the lip region, patients with BCLP show a more posterior maxilla relative to the skull base. Further studies incorporating preoperative data would enhance our understanding of this complex interplay between growth dynamics and the subsequent effects of surgical intervention.

The delayed hard palate closure has a significant influence on the anterior-posterior relation of the maxilla to the mandible [16]. Considering the uniform treatment approach in our sample this may explain why our results indicate fewer variation in the anterior-posterior dimension than in the vertical dimension. Scar tissue formation also impacts the transversal growth, but this dimension is not visualized in 2D lateral cephalograms.

Our clinical sample does not include dental casts, frontal photographs, or 3D data of the patients. Due to the comparatively low prevalence of orofacial clefts in the general population and the distribution of patients across different treatment facilities, it is difficult to collect a large and comprehensive data set. The large catchment area of our patient population, however, allows for a high degree of external validity and a large sample size, which in statistical terms is absolute and not relative to the size of the patient population [33]. Both together are advantageous in terms of the bias-variance tradeoff [43]. Our focus on lateral cephalograms sacrificed some internal validity but did not compromise the sample size, which was considerable overall and across the three groups. The sample size often poses a challenge in studies using geometric morphometrics [53]. It should be noted that the retrospective nature of this study introduces the possibility of bias in the results.

Orofacial clefts occur in various combinations and there is currently no generally accepted classification system [54]. A common method of classifying individuals into superordinate groups would facilitate comparison – this rarely seems to be acknowledged by researchers. The distribution of female and male patients in our sample reflects what has been reported for different populations: BCLP and UCLP are more common in males, while CP is more common in females [8]. Because age could be a confounding factor as growth continues into adulthood [55, 56], we only included subjects with cervical vertebral maturation stage CS5 or CS6, so that most craniofacial growth had already occurred. The lateral cephalograms were taken at a focus-film distance of 4 m, resulting in more parallel X-rays and less distortion compared with cephalograms taken at the current standard focus-film distance of 1.5 m. Nevertheless, tracing errors can complicate the reproducibility of landmark identification [57–59]. To address this issue, we chose the Bergen analysis for our cephalometric evaluations because it is well established in clinical orthodontics and is practiced daily in our clinic. Our results show very low measurement error for the variables. The Bergen analysis captures most important aspects of craniofacial morphology and was found to be equivalent to Delaire’s whole-skull analysis for viscerocranial assessment of patients with cleft [24].

The choice of treatment strategy and ultimately the choice of treatment center influences facial growth, especially in the premaxillary region [60], complicating the comparison between studies. Our findings are based on X-ray images obtained in the 1960s, illustrating treatment outcomes achieved with the knowledge and surgical techniques available at that time. Since then, our understanding of craniofacial development and growth processes has evolved, along with advancements in technology. This also may be a contributing factor in the craniofacial morphology observed in our study. Because there is still uncertainty as to whether intrinsic or extrinsic factors are primarily responsible for growth impairment [5], future research using geometric morphometrics should include unoperated subjects to further clarify the cause of growth deficiency.

To the best of our knowledge, our use of a univariate regression analysis of the first PCs on the variable cleft type represents a novel approach in the scientific literature. Thus, our results on the PCs that best represent group differences (PC4 and PC5) cannot be compared with existing publications. Other studies have focused on PC1 and PC2, which is more of a prediction model. Our focus on a risk model clarifies the research question – an important but often overlooked requirement for validity. However, the effect of cleft type on shape is weak and the PCs representing these differences still account for only 10% of the total shape variation. The methods of shape visualization after removing size, orientation and position of an object are particularly advantageous in terms of shape interpretation. Some authors have argued that the preceding Procrustes superimposition could distort shape differences because they are distributed over all landmarks rather than being depicted at the exact landmark that might differ [29, 61]. However, this so-called “Pinocchio effect” ultimately relates back to the position and orientation of the landmarks, which is not an aspect of shape in geometric morphometrics [62]. By representing the overall shape, geometric morphometrics provide a more realistic insight into cleft-specific craniofacial morphology. Nevertheless, this method is not yet widely used to assess patients with cleft, making it difficult to compare our results with other studies. This limitation also highlights the need for our study and further studies using geometric morphometrics. A thorough understanding of cleft anatomy and the effects on craniofacial morphology and dentofacial relation is essential to providing patients with cleft with treatments tailored to their specific needs.

## Conclusion

Cleft type has a statistically significant but weak effect on craniofacial morphological variability in patients with non-syndromic orofacial clefts, mainly in the vertical dimension. Greater shape variation is observed in subjects with cleft types that have a greater extent of the defect.

This study contributes to the literature particularly due to our risk model approach in lieu of a prediction model. Further studies could focus on applying this approach to data from 3D scans, photographs, and dental casts.

## Data Availability

No datasets were generated or analysed during the current study.
